# Implementing Heat-Stable Carbetocin for Postpartum Haemorrhage Prevention in Low-Resource Settings: A Rapid Scoping Review

**DOI:** 10.3390/ijerph19073765

**Published:** 2022-03-22

**Authors:** Nguyen Toan Tran, Sarah Bar-Zeev, Willibald Zeck, Catrin Schulte-Hillen

**Affiliations:** 1 Australian Centre for Public and Population Health Research, Faculty of Health, University of Technology Sydney, Sydney, NSW 2007, Australia; 2 Faculty of Medicine, University of Geneva, 1211 Geneva, Switzerland; 3 United Nations Population Fund, Technical Division, New York, NY 10158, USA; bar-zeev@unfpa.org (S.B.-Z.); zeck@unfpa.org (W.Z.); 4 United Nations Population Fund, Humanitarian Office, 1202 Geneva, Switzerland; schulte-hillen@unfpa.org

**Keywords:** heat-stable carbetocin, postpartum haemorrhage, prevention, health system, low-resource settings

## Abstract

Heat-stable carbetocin (HSC), a long-acting oxytocin analogue that does not require cold-chain transportation and storage, is effective in preventing postpartum haemorrhage (PPH) in vaginal and caesarean deliveries in tertiary-care settings. We aimed to identify literature documenting how it is implemented in resource-limited and lower-level maternity care settings to inform policies and practices that enable its introduction in these contexts. A rapid scoping review was conducted with an 8-week timeframe by two reviewers. MEDLINE, EMBASE, Emcare, the Joanna Briggs Institute Evidence-Based Practice Database, the Maternity and Infant Care Database, and the Cochrane Library were searched for publications in English, French, and Spanish from January 2011 to September 2021. Randomized and non-randomized studies examining the feasibility, acceptability, and health system considerations in low-income and lower-middle-income countries were included. Relevant data were extracted using pretested forms, and results were synthesized descriptively. The search identified 62 citations, of which 12 met the eligibility criteria. The review did not retrieve studies focusing on acceptability and health system considerations to inform HSC implementation in low-resource settings. There were no studies located in rural or lower-level maternity settings. Two economic evaluations concluded that HSC is not feasible in terms of cost-effectiveness in lower-middle-income economies with private sector pricing, and a third one found superior care costs in births with PPH than without. The other nine studies focused on demonstrating HSC effectiveness for PPH prevention in tertiary hospital settings. There is a lack of evidence on the feasibility (beyond cost-effectiveness), acceptability, and health system considerations related to implementing HSC in resource-constrained and lower-level maternity facilities. Further implementation research is needed to help decision-makers and practitioners offer an HSC-inclusive intervention package to prevent excessive bleeding among pregnant women living in settings where oxytocin is not available or of dubious quality.

## 1. Introduction

Obstetric haemorrhage, particularly postpartum haemorrhage (PPH), is responsible for more than one quarter of all maternal deaths worldwide [1]. The overwhelming majority of these deaths continue to occur in low- and middle-income countries, particularly those impacted by humanitarian disasters [2].

Uterine atony accounts for around two-thirds of PPH cases, which can also be caused by trauma to the genital tract, retained placental tissue, or maternal bleeding disorders. Most deaths from PPH could be avoided through the active management of the third stage of labour (AMTSL). AMTSL involves the prophylactic administration of a uterotonic agent prior to placental delivery, as well as delayed cord clamping and controlled traction of the umbilical cord (in settings where skilled birth attendants are available) [3]. The World Health Organization (WHO) recommends the administration of a prophylactic uterotonic to all women directly after birth to prevent PPH. This is the most critical AMTSL component with oxytocin (10 IU, IM/IV) being the uterotonic of choice in settings where multiple uterotonic agents are available, according to WHO recommendations [3,4,5].

Heat-stable carbetocin (HSC), a long-acting synthetic oxytocin analogue recommended only for PPH prevention, was recently added to the core list of reproductive health medicines of the WHO Model List of Essential Medicines (2019 edition) [6]. HSC does not require cold-chain transport and storage that impede oxytocin use—an operational advantage in low-resource settings. Several studies and systematic reviews have demonstrated the effectiveness of HSC, including in low-income countries. Notably, the randomized controlled trial (RCT) published in 2018 by Widmer et al. demonstrated the noninferiority of HSC compared to oxytocin to prevent blood loss of at least 500 mL or the use of additional uterotonics in vaginal births [7]. This large hospital-based trial was undertaken in ten countries (Argentina, Egypt, India, Kenya, Nigeria, Singapore, South Africa, Thailand, the United Kingdom, and Uganda—the single low-income country according to the 2021 World Bank list) [8]. Gallos et al., in their 2018 Cochrane review on uterotonics for PPH, identified that Ergometrine plus oxytocin combination, carbetocin, and misoprostol plus oxytocin combination may have some additional desirable effects compared with the current standard oxytocin. The two combination regimens, however, are associated with significant side effects. Carbetocin may be more effective than oxytocin for some outcomes without an increase in side effects. The review incorporated 196 trials mostly on hospital-based vaginal births across 53 countries, including high-, middle-, and low-income countries [9]. Voon et al. concluded in their 2018 meta-analysis of RCTs that HSC is effective in reducing the use of additional uterotonics and decreasing PPH and transfusion when used during caesarean deliveries [10]. Among the included studies, none were conducted in low-income countries; one by El Behery et al. was from Egypt was conducted in a lower-middle-income country [11].

HSC has been relatively costly compared to other uterotonics. Recent subsidization efforts for the public sector of low-income and middle-income countries have resulted in competitive prices. The 2021 Product Catalogue of the United Nations Population Fund (UNFPA) listed a price of USD 0.413 per HSC ampoule compared to USD 0.334 per oxytocin ampoule [12]. Against this background, the WHO recommends HSC in situations when (i) oxytocin is unavailable or of dubious quality, (ii) there is no cold transportation and storage capability, (iii) its cost is comparable to that of other effective uterotonics, and (iv) there is skilled health personnel to inject it [4].

To facilitate the implementation of HSC in low-resource settings, policymakers and program managers need additional information beyond its effectiveness. This includes the feasibility of its use, its acceptability by women and providers, and other health system requirements, such as policy change, staff capacitation, or procurement and storage considerations. A synthesis of existing experience from low-resource settings may offer helpful guidance. To the best of our knowledge, there are no published reviews on this research question. As such, this scoping review aimed to summarize HSC implementation experience from low-resource settings.

## 2. Materials and Methods

We conducted a rapid scoping review of the literature to synthesize recent practices and health system considerations to inform HSC roll-out in low-resource settings. A rapid review is a type of knowledge synthesis in which researchers abridge or skip parts of the systematic review process to obtain information in a shortened timeframe [13]. Scoping reviews are intended to give researchers, decision-makers, and practitioners an overview of a topic to identify the main ideas, evidence types, and knowledge gaps within an emerging topic [14]. The PRISMA statement was used to report the results (see Supplementary Materials) [15].

### 2.1. Protocol

We developed a protocol but did not register it because of limited time (see Supplementary Materials).

### 2.2. Literature Search Strategy

We searched the following five bibliographic databases via the Ovid research platform to find relevant documents: MEDLINE, EMBASE, Emcare, the Joanna Briggs Institute Evidence-Based Practice (JBI EBP) Database, and the Maternity and Infant Care Database (MIDIRS). We also searched the Cochrane Library. The leading search concepts were carbetocin, postpartum haemorrhage, and developing countries, a Medical Subject Heading (MeSH) term in MEDLINE, and a proxy for low-resource settings. The search was limited to documents in English, French, and Spanish published between 1 January 2011 and 15 September 2021.

Based on our knowledge of the literature, we conducted an initial exploratory search, which did not yield sufficient and expected results, including work by Widmer et al. [7]. Consequently, we revised our strategy in two steps. First, we expanded the concept of developing countries to include all the countries of the low-income and lower-middle-income groups as defined by the World Bank (see Supplementary Materials) [8]. Second, we kept the search open to carbetocin effectiveness studies done in low-income and lower-middle-income countries, hypothesizing that such articles may still offer insights into the operational concepts of interest (levels of care, feasibility, acceptability, health system environment). Supplementary Materials contains the final MEDLINE search strategy. We exported the search results into EndNote for duplicate removal.

### 2.3. Eligibility Criteria

Titles and abstracts were reviewed at the first stage of screening. Potential papers were retrieved and evaluated for inclusion using the criteria in Table 1, which guided the final selection of full-text publications. The eligibility criteria were defined using the population, concept, and context (PCC) framework in addition to study design and publication type. The PCC framework is recommended by the JBI for Scoping Reviews as a more suitable alternative to the PICO mnemonic (population, intervention, comparator, and outcome) recommended for systematic reviews [16,17].

### 2.4. Study Selection and Data Abstraction

Non-duplicated articles were imported into JBI SUMARI (System for the Unified Management, Assessment, and Review of Information) for further screening [18]. Titles and abstracts were assessed for eligibility, and the full texts of eligible articles were reviewed. The data of included studies were extracted using tables developed a priori and pilot-tested on a random sample of two articles. One table included study characteristics (e.g., first author, publication year, location, aim, method, study population, intervention type and outcome measures, and relevant findings). Another table focused on concepts relevant to our research question, including country income classification, levels of care (basic (BEmOC) vs. comprehensive (CEmOC) emergency obstetric care), and outcomes of interest (feasibility, acceptability, and effectiveness, as it was often also a feature of the retrieved articles). Equally important were health system considerations, which were drawn from the WHO framework of the six-health system building blocks, i.e., (i) government and policy alignment, (ii) procurement channels and commodity security, (iii) health staff awareness, motivation, and training, (iv) service delivery, (v) health information system, and (vi) financing [19]. Two reviewers performed independently all the steps of screening and data extraction; discrepancies were resolved by a third reviewer.

### 2.5. Methodological Quality Appraisal

In accordance with recommendations on the conduct of scoping reviews, there was no appraisal of the methodological quality of the included articles [20].

### 2.6. Synthesis

The review findings are presented descriptively with the aid of tables on study characteristics and relevant program considerations.

## 3. Results

### 3.1. Quantity of Research Available

**Figure 1:**
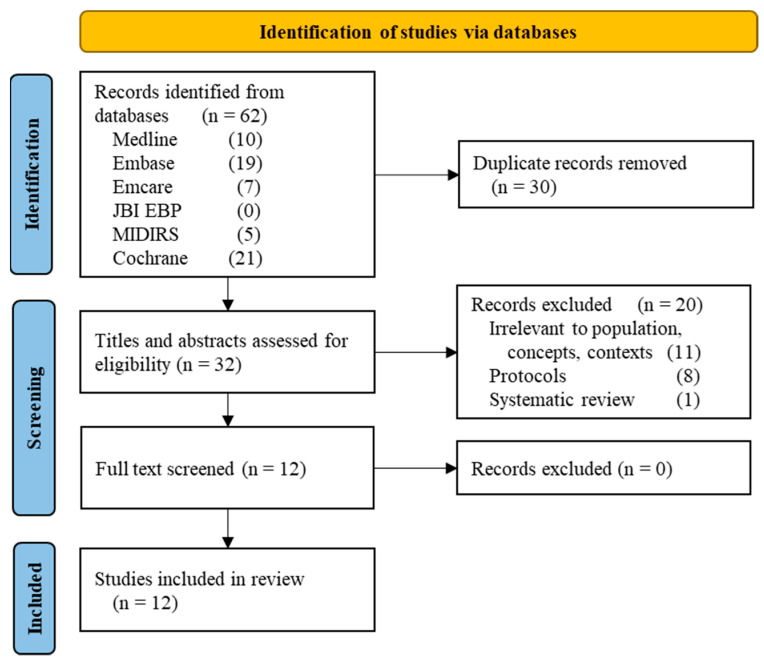
Study flow chart.

The search of electronic databases found 62 citations, of which 30 were duplicates (Figure 1). From the remaining 32, based on titles and abstracts, 20 were removed because they were systematic reviews (1) (Voon et al., see Background), protocols (8), or irrelevant to our subject (11). A total of 12 studies were identified for full-text screening text. The full text of Akhter et al. could not be retrieved despite searches in online platforms and the libraries of the University of Geneva, Switzerland, and the University of Technology Sydney Australia [21]. Moosivand et al. and Farhad et al. were conference abstracts, which appeared to have no subsequent full-article publication [22,23]. Due to their relevance and detailed abstracts, all three studies were included in the final selection, which encompassed a total of 12 references. Key results are presented narratively according to the criteria outlined in Table 1 (study design, PCC). The characteristics of the included studies are summarized in Table 2.

### 3.2. Study Design

#### 3.2.1. Randomized Controlled Trials

The RCTs by Elbohoty et al. [24], Farhad et al. [23], Kabir et al. [25], Maged et al. [26], and Nahaer et al. [27] were based in one study site, Taheripana et al. [28] in two sites in the same country (Iran), and Widmer et al. [7] in 23 sites in ten countries. Randomization is unknown for the study by Akhter et al. [21].

#### 3.2.2. Non-Randomized Trials

Razzaque et al. [29] followed up women in a prospective single-centre study.

#### 3.2.3. Economic Analyses

Briones et al. assessed the budget impact of HSC relative to oxytocin in vaginal and caesarean births using a decision tree over a six-week time horizon and with the cost perspective of a major government maternity hospital in Manila, the Philippines [30]. Moosivand et al. also used a decision tree model applied to the Iranian context but over a one-year timeframe and with a human capital approach to estimate indirect costs [31]. Theunissen et al. collected the costs of direct hospital care (stay, medications, transfusions) of women who received oxytocin or HSC in India, Kenya, Nigeria, and Uganda [32]. Farhad et al. mentioned uterotonic costs per patient in the conclusion of their effectiveness study but did not detail the methods used for this analysis [23].

**Table 2:** Characteristics of included studies.

Study & Year	Location	Aim	Method/Design	Study Population and Sample Size	Intervention Type & Outcome Measures	Relevant Findings
Akhter et al., 2018 [21] (Full text not available)	Sir Salimulla Medical College Hospital, Dhaka, Bangladesh	To compare the effectiveness of HSC and oxytocin in the management of 3rd stage of labour in preventing PPH post vaginal delivery	Two-arm clinical trial in hospital CEmOC No information on randomization	300 pregnant women undergoing normal vaginal delivery Gestational age not mentioned	HSC 100 mcg IV vs. oxytocin 10 IU IV Primary outcome: PPH	23 (15.3%) and 31 (20.7%) patients in HSC and oxytocin groups respectively. A significantly higher number of patients was treated with balloon catheter in oxytocin group (77.4%) than HSC group (39.1%). Thirteen (41.9%) patients in oxytocin group and 4 (17.4%) patients in HSC group needed ICU treatment.
Briones et al., 2020 [30]	Philippines	To determine the cost-effectiveness and budgetary impact of HSC against oxytocin in the Philippines	Cost-utility analysis using a decision tree to compare the costs (direct medical and non-medical, indirect) and outcomes of HSC vs. oxytocin for PPH prophylaxis in women with either vaginal delivery or c-section in a six-week time horizon	180 women with vaginal birth (100) or c-section (80) Direct medical costs collected from women without complication, with additional treatment dose, blood transfusion, and hysterectomy	Budget impact analysis using the hospital billing records of a tertiary level birthing hospital in Manila Primary outcome: incremental cost-effectiveness ratio (ICER) using a USD 2895 per quality adjusted life year (QALY)	HSC was not cost-effective given the listed price of HSC at 18 USD. Given a societal perspective, the ICER values of USD 13,187 and over USD 40,000 per QALY gained were derived for c-section and vaginal delivery, respectively. The five-year total budget impact of a drug mix of HSC and oxytocin was USD 25.54 million (4.23 million for c-section and 21.31 million for vaginal delivery) compared with “only oxytocin” scenario.
Elbohoty et al., 2016 [24]	University maternity hospital in Cairo, Egypt	To compare the effectiveness and safety of HSC, misoprostol, and oxytocin for post c-section PPH prevention	Double-blind randomized controlled trial enrolled	263 at-term pregnant women with singleton pregnancy scheduled for an elective c-section	HSC 100 mcg IV vs. misoprostol 400 mcg SL vs. oxytocin 10 IU IV Primary outcome: uterine atony necessitating additional uterotonics	HSC was comparable to oxytocin and superior to misoprostol in the prevention of uterine atony after c-section. Further uterotonics were needed for the treatment of 5 (6%) patients who were treated with HSC, 20 (22%) patients treated with misoprostol, and 11 (13%) patients treated with oxytocin. In the prevention of uterine atony, HSC was comparable with oxytocin (RR 0.41, 95% CI 0.14–1.25) and superior to misoprostol (RR 0.21, 95% CI 0.07–0.58).
Farhad et al., 2017 [23] (Conference abstract)	Anwar Khan Modern Medical College and Hospital, Dhaka, Bangladesh	To compare the effectiveness and safety of HSC over oxytocin in the active management of the 3rd stage of labour post c-section	Double-arm randomized controlled trial Blinding not mentioned	200 at-term pregnant women undergoing elective or emergency c-section	HSC 100 mcg IV vs. oxytocin 10 IU IV Primary outcome: primary PPH, safety and efficacy, need for additional uterotonic therapy, immediate blood transfusion, adverse effects and cost-effectiveness	HSC can be considered a good alternative to oxytocin in the active management of the third stage of labour in caesarean section: PPH occurred in 8% of women vs. 2% of the HSC group. Additional uterotonics were needed for 10% of women in the oxytocin group vs. 2% in the HSC group. Immediate blood transfusion was needed for 8% in the oxytocin group vs. 4% in the HSC group. Fluid overload occurred in 8% of women in the oxytocin group but did not in the HSC group. Adverse effects were more observed in the oxytocin group. Average uterotonic cost per patient in the HSC group was less in comparison with the oxytocin group (but no mention of HSC price).
Kabir et al., 2015 [25]	Institute of Child and Mother Health, Dhaka, Bangladesh	To evaluate the efficacy and safety of HSC in comparison to oxytocin in the active management of third stage of labour following vaginal delivery	Two-arm randomized-controlled trial Blinding not mentioned	94 at-term pregnant women admitted for vaginal delivery	HSC 100 mcg IV vs. oxytocin 10 IU IM Primary outcome: blood loss in 24 h, primary PPH, massive blood loss, need of fundal massage, need for additional uterotonic therapy, blood transfusions	HSC appears to be an effective new drug in the active management of 3rd stage of labour in vaginal delivery. A single dose of 100 mcg IV HSC is more effective than oxytocin for maintaining adequate uterine tone, less blood loss and preventing postpartum bleeding in women undergoing vaginal delivery: primary PPH in 6.4% in oxytocin group vs. none in HSC group. Massive blood loss in 8.5% women of oxytocin group. Further fundal massage, immediate blood transfusion and additional uterotonics were not needed in HSC group. In oxytocin group, fundal massage required in 10.6% of women, blood transfusion in 6.4% and additional uterotonics in for 10.6% women.
Maged et al., 2020 [26]	Kasr Al Ainy Hospital, Cairo, Egypt	To compare effectiveness and safety of HSC and misoprostol for PPH prevention among low-risk women with vaginal delivery	Two-arm open-label randomized-controlled trial	150 women with singleton pregnancy of 36–40 weeks, low risk of PPH, and admitted for vaginal delivery	HSC 100 mcg IV vs. misoprostol (2 rectal tablets totalling 800 mcg) Primary outcome: need for additional uterotonics	Among low-risk women, HSC seems to be a better alternative to misoprostol for active management of the 3rd stage of labour, reducing blood loss and use of additional uterotonic drugs: the HSC group had significantly less blood loss (*p* < 0.001), shorter third stage (*p* < 0.001), and less need for additional uterotonics (*p* = 0.013) or uterine massage (*p* = 0.007). The two drugs were hemodynamically safe. Haemoglobin levels after delivery were comparable in the two groups (*p* = 0.475). Adverse effects were more common in the misoprostol group (*p* < 0.001)
Moosivand et al., 2016 [22] (Conference abstract)	Iran	To assess the cost-utility of HSC versus Oxytocin in the context of Iran	Cost-utility analysis Human capital approach applied for estimating indirect costs	Model population not mentioned	Cost utility of HSC vs. oxytocin Primary outcome: Incremental cost-effectiveness ratio (ICER)	In the highest price scenario for oxytocin and other costs in the private sector, the total extra cost of HSC was not significant because of the small number of candidates for HSC. HSC is not cost-effective in other scenarios.
Nahaer et al., 2020 [27]	Rangpur Medical College and Hospital, Rangpur, Bangladesh	To assess the efficacy and safety of HSC vs. oxytocin for PPH prevention in c-section	Two-arm open-label randomized-controlled trial	100 women with singleton pregnancy undergoing c-section Not mentioned: gestational age, elective vs. emergency c-section	HSC 100 mcg IV vs. oxytocin 10 IU IM Primary outcome: primary PPH, massive blood loss, need for additional uterotonic drug	Oxytocin group: PPH in 8%, massive blood in 6%, blood transfusion needed in 20%, additional uterotonic needed for 36%, vs. HSC group: no PPH, no massive blood, 2% needed immediate blood transfusion, 4% patients needed additional uterotonics. There were no major adverse effects observed in both the groups.
Razzaque and Khan 2020 [29]	Bagerhat Sadar Hospital, Bangladesh	To assess the efficacy and safety of HSC for the prophylaxis of PPH during c-section	Open label single arm clinical trial in hospital CEmOC	90 women with term singleton pregnancy undergoing c-section for cephalopelvic disproportion, malpresentation, previous c-section, foetal distress, very low birth weight and failed induction of labour	HSC 100 mcg IV (no comparator) Primary outcome: PPH defined as blood loss from genital tract of 1000 mL or more within 24 h post c-section	HSC appeared effective for primary PPH prevention in c-section: 4.4% (4/90) had PPH, with 3.3% (3/90) with massive blood loss (>50% of circulating blood volume within 3-h) requiring additional uterotonics.
Taheripanah et al., 2018 [28]	Two university-based hospitals in Tehran, Iran	To compare the use of HSC and oxytocin for PPH prevention in c-section	Prospective double-blind two-arm randomized controlled clinical trial	220 at-term pregnant women requiring emergency c-section	HSC 100 mcg IV vs. 30 IU IV infusion of oxytocin during 2 h after delivery of placenta. Primary outcome: PPH requiring additional uterotonic drugs, bleeding volume, and haemoglobin level	HSC is a good alternative modality to conventional uterotonic agents such as oxytocin for the PPH prevention after c-section. Difference between HSC vs. oxytocin regarding haemoglobin drops (1.01 versus 2.05, *p* = 0.01), bleeding volume (430.68 mL versus 552.6 mL, *p* < 0.001), uterine massages frequency (3.7 versus 4.26, *p* < 0.001), and uterine height at 2, 4, and 24 h (*p* < 0.001). Oxytocin side effects were significantly higher in comparison with the HSC except pruritus which was observed in 27% of patients in the HSC versus no cases in the oxytocin group.
Theunissen et al., 2021 [32]	Nine tertiary care facilities in India, Kenya, Nigeria, and Uganda	To assess the costs of care of women receiving different preventative uterotonics and with PPH and without PPH	Assessment of costs of direct hospital care of women who received oxytocin or HSC (provided at low prices) for prevention of PPH	2966 women with PPH (1481, oxytocin: 742, HSC: 739) and without PPH (1485, oxytocin: 741, HSC: 744)	Cost-analysis of HSC 100 mcg vs. oxytocin 10 IU for hospital stay, PPH interventions, staff labour during intervention, transfusions, and medications, including additional uterotonics. Primary outcome: Difference in cost of care at a facility level between women who experienced a PPH event and those who did not.	Increased cost of care for PPH up to 2.8 times that for a birth without PPH: the mean cost of care of a woman experiencing PPH in the study sites in India, Kenya, Nigeria, and Uganda exceeded the cost of care of a woman who did not experience PPH by between 10% (Uganda) and 180% (Nigeria). There was a large variation in cost across hospitals within a country and across countries. PPH cases were associated with increased interventions during labour, such as augmentation, instrumental delivery, and episiotomy and tears (it is likely that over-medicalization was also associated with increased costs). Authors noted: “Although initially we intended to compare the costs between oxytocin and heat stable carbetocin, we did not include this analysis in the main body of the paper because the clinical effects were similar with both (this information is available in Additional file 1: Annex)”. Note: HSC and oxytocin donated for the study.
Widmer et al., 2018 [7]	23 hospital sites in 10 countries: Argentina, Egypt, India, Kenya, Nigeria, Singapore, South Africa, Thailand, Uganda, UK	To compare HSC with oxytocin	Randomized, double-blind, noninferiority trial with HSC and oxytocin donated for the study	29,645 women with term singleton pregnancy and vaginal birth	HSC 100 mcg vs. oxytocin 10 IU IM immediately after vaginal birth Primary outcome: proportion of women with blood loss of at least 500 mL or the use of additional uterotonic agents, and the proportion of women with blood loss of at least 1000 mL	HSC was noninferior to oxytocin for the prevention of blood loss of at least 500 mL or the use of additional uterotonic agents. Noninferiority was not shown for the outcome of blood loss of at least 1000 mL; low event rates for this outcome reduced the power of the trial. The use of additional uterotonic agents, interventions to stop bleeding, and adverse effects did not differ significantly between the two groups.

HSC: heat-stable carbetocin; IM: intramuscular; IU: international unit; IV: intravenous; PPH: postpartum haemorrhage; SL: sublingual.

### 3.3. Patient Population

#### 3.3.1. Randomized Controlled Trials

All the trials enrolled women with a singleton pregnancy. Most participants were recruited from 37 weeks of pregnancy and above except in Maged et al., which started at 36 weeks [26]. Gestational age was not mentioned in Akhter et al. [21] and Nahaer et al. [27]. Four trials involved women with vaginal delivery (Widmer et al., Akhter et al., Kabir et al., and Maged et al.) [7,21,25,26]. Four trials involved women undergoing c-section, electively (Elbohoty et al. [24]), in emergency (Taheripana et al. [28]), or both elective and in emergency (Farhad et al. [23])—Nahaer et al. did not report this information [27]. All the studies were relatively small in size (90 to 300 participants) except for Widmer et al., which recruited 29,645 participants.

#### 3.3.2. Non-Randomized Trials

Razzaque et al. enrolled 90 at-term singleton-pregnancy women undergoing elective or emergency c-section [29].

#### 3.3.3. Economic Analyses

The economic modelling of Briones et al. included 180 women with vaginal birth (100) or c-section (80) [30]. Moosivand et al. did not detail the modelling population [31]. Theunissen et al. analysed the data of a total of 2966 women: 1481 had PPH and received either preventive oxytocin (742) or HSC (739), and 1485 without PPH (preventive oxytocin: 741, HSC: 744) [32].

### 3.4. Context

All the trials were based in large tertiary referral hospitals providing CEmOC. So were the three cost-analysis modelling studies. None took place in BEmOC or rural facilities. Out of the twelve studies, only two were in a low-income country, i.e., Uganda (Widmer et al. [7], Theunissen et al. [31]). In fact, the latter study was a cost analysis of the former but using data from three lower-middle-income countries (India, Kenya, and Nigeria) in addition to data from Uganda. These countries were part of the larger ten-country study by Widmer et al., which also included another lower-middle-income country (Egypt), three upper-middle-income countries (Argentina, South Africa, and Thailand), and two high-income countries (Singapore and the United Kingdom) [7]. The remaining studies were based overwhelmingly in Bangladesh (5: Akhter et al. [21], Farhad et al. [23], Kabir et al. [25], Nahaer et al. [27], and Razzaque et al. [29]), followed by Egypt (2: Elbohoty et al. [24] and Maged et al. [26]), Iran (2: Moosivand et al. [32] and Taheripana et al. [28]), and the Philippines (Briones et al. [30]).

### 3.5. Concepts

The main concepts of the included studies are summarized in Table 3. Except for the three economic analyses, none of the other studies addressed the health system components to consider for HSC implementation. In the same line, there was limited information available on the feasibility of implementing HSC in low-resource settings except economic feasibility (see below). There was also no specific investigation of acceptability by providers or women (maybe apart from the side effects of HSC vs. oxytocin reported in the effectiveness studies).

#### 3.5.1. Randomized and Non-Randomized Controlled Trials

The effectiveness trials compared HSC 100 mcg IV with oxytocin 10 IU IV (30 IU IV in Taheripana et al. [28]) except for the following studies: Maged et al. [26] used misoprostol 800 mcg per rectum as a comparator and found it to be less effective for low-risk vaginal births; Elbohoty et al. [24] used both oxytocin and misoprostol 400 mcg sublingually as comparators and concluded that HSC was comparable to oxytocin but superior to misoprostol for PPH prevention in c-section; and Razzaque et al. [29] did not have a comparator and concluded that HSC appeared to be effective for PPH prevention in c-section. Overall, the HSC vs. oxytocin trials concluded that HSC is an effective alternative to oxytocin for vaginal and c-section deliveries.

#### 3.5.2. Economic Analyses

In the Philippines, Briones et al. found that HSC was not cost-effective for PPH prevention in vaginal and caesarean births, given the listed price of HSC at USD 18 [30]. In Iran, Moosivand et al. concluded that HSC was not cost-effective, except in the scenario with the highest price of oxytocin and other costs in the private sector [31]. Theunissen et al. used a public sector price for HSC, which was competitive to that of oxytocin [32]. Although the authors initially intended to compare the costs between HSC and oxytocin, they did not present this comparison in the main body of the paper because the clinical effects were similar with both. Instead, the analysis focused on the increased cost of PPH care and found its cost up to 2.8 times that of births without PPH, ranging from 10% (Uganda) to 180% (Nigeria). In Bangladesh, Farhad et al. found that the average uterotonic cost per c-section patient in the HSC group was less in comparison with the oxytocin group [23]. However, there was no mention of the HSC price (presumably low public sector price) and the methodology used for the analysis.

## 4. Discussion

This scoping review included three economic evaluations, eight RCTs, and one non-randomized trial. The only low-income study country was Uganda, while the other study countries were lower-middle-income and more developed economies. The effectiveness studies showed HSC to be an effective alternative to oxytocin and more effective than misoprostol in singleton vaginal and caesarean births in tertiary care CEmOC settings. The economic analyses determined that HSC is not cost-effective in lower-middle-income economies with private sector pricing, and care costs are superior in births with PPH than without PPH in low-income and lower-middle-income countries. The review did not retrieve studies focusing on acceptability and health system considerations to inform HSC implementation in low-resource settings. Notably, there was no study located in rural, BEmOC, or lower-level maternity settings.

With the concentration of global maternal deaths in the least developed countries, including those affected by fragility and humanitarian crises, it is important to pilot and research the impact of HSC in such settings [2]. In this review, Uganda, with a maternal mortality ratio of 375 per 100,000 live births in 2017, stands out as the single low-income research country, and Bangladesh, with an MMR of 173 per 100,000 live births in 2017, was the study site for five out of the nine effectiveness studies [2]. All studies were conducted in urban tertiary care CEmOC settings. It is, however, in resource-constrained and BEmOC contexts, where power shortages are more likely to threaten the reliable cold-chain storage of oxytocin, making HSC a critical alternative to oxytocin for PPH prevention [33]. It is therefore strategic to pilot and evaluate the implementation of HSC in lower-level maternity settings that do not have a reliable cold-chain storage system.

The results of the effectiveness studies are aligned with the evidence gathered globally on HSC for vaginal and c-section births, including the Cochrane review by Gallos et al. and the systematic review on HSC in caesarean deliveries by Voon et al. [9,10]. Owing to existing evidence, the review of HSC effectiveness in low-resource settings was not the primary objective of our scoping working. We had hoped that the full-text review of the identified effectiveness studies would have offered insights into implementation considerations relevant to low-resource settings. We did not find it to be the case and are aware that research by Akhter et al. [21] and Farhad et al. [23] may contain pertinent information. There were unfortunately no available full-text papers. In particular, Farhad et al. [23] might offer some understanding of HSC economic feasibility as briefly mentioned at the end of its related conference abstract.

With regard to the economic feasibility of HSC, the lack of cost-effectiveness at private sector pricing found in the Philippines and Iran, two lower-middle-income countries, reflects to some extent the results from an economic analysis done in 2018 by Gil-Rojas et al. in the context of Columbia, an upper-middle-income country [34]. The study used only direct medical costs with private sector HSC pricing and a time horizon of one year. It concluded that HSC was the dominant alternative in the prevention of PPH compared to oxytocin in case of elective caesarean delivery, but not in vaginal delivery where both uterotonics had similar effectiveness. Given the similar clinical effects of HSC in vaginal delivery, Theunissen et al. did not compare the costs between HSC, which was obtained at public sector price for their research, and oxytocin. The study documented the somewhat unsurprisingly increased cost of vaginal births complicated by PPH, which had a wide range across countries (up to 2.8 times in Nigeria). The authors noted that overmedicalization of labour and delivery, such as labour augmentation, episiotomy, and instrumental delivery, which is documented in both high- and low-resource countries [35,36], likely contributed to the increased costs in managing women experiencing PPH irrespective of the uterotonics used. The scoping review did not identify economic evaluations done in BEmOC and rural settings. Owing to the facilitated access to the public-sector price of HSC, such evaluations would be helpful, especially to compare the cost-effectiveness of HSC with misoprostol as both uterotonics are not cold-chain dependent and, therefore, suitable for settings lacking reliable cold chain capacity. HSC requires staff skilled to administer this via an injection while misoprostol does not, as it can be administered through the oral, buccal, vaginal, or anal routes. This is an important operational aspect to consider in low-resource settings and settings with skilled staff shortages [37]. Introducing HSC would require not only initial capacity building, particularly of midwives as the main providers of care at birth, but also ongoing support of providers through mentorship and supportive supervision following training to ensured high quality of care.

Overall, the scoping review offered limited information on the health system components that decision-makers and practitioners might want to consider when planning and implementing PPH prevention interventions that include HSC [38]. For instance, there was no useful information on how to (i) work with policymakers and integrate HSC into health policies and technical guidance, (ii) procure HSC and secure its availability, (iii) develop health staff capacity and motivate them to use HSC, (iv) integrate HSC into patient records and the overall health information system, or (v) deliver PPH prevention interventions that integrate HSC.

### 4.1. Implications for Research and Practice

Therefore, further implementation research in low-resource and BEmOC settings would be helpful to explore health system strategies to promote the systematic uptake of evidence-based practices that are available at higher levels of care, such as HSC for PPH prevention [39]. Methodologically rigorous implementation research would allow policymakers, managers, and practitioners to answer the following question: HSC effectively prevents PPH in hospital-based tertiary referral settings; how can it be implemented in lower-level maternity care settings, where oxytocin is not available or of dubious quality? Pilot projects should therefore include robust implementation sciences to document methodically how the proposed approaches are implemented and the operational barriers and enablers they encounter [38]. The projects should judiciously be an opportunity to refresh clinicians’ PPH knowledge and skills, in addition to training them on HSC as an effective option in the overall PPH prevention toolkit. The design, implementation, and study of such pilots should involve key stakeholders, including women, clinicians, managers, and policymakers. Such a multi-stakeholder engagement is essential for conducting participatory assessments of needs and resources, as well as developing a project that is safe, acceptable, feasible, and sustainable for all parties involved. It may be advantageous to build on the experience of some of the low-resource countries, such as Bangladesh and Egypt, as they appear to have extensively studied HSC—however, at the tertiary care level. Researchers from these study countries should be encouraged to extend their research scope to include BEmOC facilities in rural and underserved settings.

### 4.2. Limitations

Our scoping review methods have limitations, many of which are inherent to the very nature of scoping reviews, as these concentrate on identifying knowledge gaps and implications for decision-making in addition to guiding future research [20]. Therefore, the shorter timescale for literature searches, article retrieval, and assessment may have induced biases. Limiting our search to English, French, and Spanish articles and accelerating the data extraction procedure may have omitted important studies and data. We also did not register our protocol and cannot comment on the scientific robustness of the included studies as their methodological quality was not examined. Due to time limitations, the lists of references of the articles may not have been thoroughly scanned and we did not contact authors for additional information.

## 5. Conclusions

HSC has the advantage of not requiring cold transportation and storage, it is proven effective in preventing PPH in vaginal and caesarean births in tertiary care level settings, and it has competitive pricing in the public sector of low-income economies. There is, however, a lack of evidence on the feasibility, acceptability, and health system and health service adaptations required for its implementation in resource-constrained and lower-level maternity care facilities. We recommend further implementation research to investigate these questions and help decision-makers and practitioners offer an HSC-inclusive intervention package to prevent excessive bleeding among pregnant women living in under-resourced settings.

## Figures and Tables

**Table 1:** Eligibility criteria.

Study design and publication type	Randomized controlled trials; non-randomized trials; peer-reviewed (no grey literature)
Timeline	Published between 1 January 2011 and 15 September 2021
P (population)	Women who had a vaginal or caesarean birth
C (concept)	Postpartum haemorrhage; feasibility; acceptability; health system considerations
C (context)	Low-income countries; lower-middle-income countries

**Table 3:** Contexts and main concepts of included studies.

Study & Year	Country		Level of Care		Outcome of Interest			Health System Environment					
	Low-Income	Lower-Middle-Income	BEmOC	Hospital CEmOC	Feasibility	Acceptability	Effectiveness	Governance & Policy Alignment	Procurement &Commodity Security	Health Staff Awareness, Motivation & Training	Service Delivery	Health Information System	Financing
Akhter et al., 2018 [21]	-	✓	-	✓	-	-	✓	-	-	-	-	-	-
Briones et al., 2020 [30]	-	✓	-	✓	-	-	-	-	-	-	-	-	✓
Elbohoty et al., 2016 [24]	-	✓	-	✓	-	-	✓	-	-	-	-	-	-
Farhad et al., 2017 [23]	-	✓	-	✓	✓	-	✓	-	-	-	-	-	✓
Kabir et al., 2015 [25]	-	✓	-	✓	-	-	✓	-	-	-	-	-	-
Maged et al., 2020 [26]	-	✓	-	✓	-	-	✓	-	-	-	-	-	-
Moosivand et al., 2016 [22]	-	✓	?	?	✓	-	-	-	-	-	-	-	✓
Nahaer et al., 2020 [27]	-	✓	-	✓	-	-	✓	-	-	-	-	-	-
Razzaque and Khan 2020 [29]	-	✓	-	✓	-	-	✓	-	-	-	-	-	-
Taheripanah et al., 2018 [28]	-	✓	-	✓	-	-	✓	-	-	-	-	-	-
Theunissen et al., 2021 [32]	✓	✓	-	✓	✓	-	-	-	-	-	-	-	✓
Widmer et al., 2018 [7]	✓	✓	-	✓	-	-	✓	-	-	-	-	-	-

✓: concept found in article; -: concept not found in article; ?: concept not mentioned; BEmOC: basic emergency obstetric care; CEmOC: comprehensive emergency obstetric care.

## Data Availability

Not applicable.

## Supplementary Materials

The following supporting information can be downloaded at: https://www.mdpi.com/article/10.3390/ijerph19073765/s1. Supplementary materials contains the PRISMA statement, the protocol, low-income and lower-middle-income groups as defined by the World Bank, and the MEDLINE search strategy.
