# Supplementary material for: Implementing Heat-Stable Carbetocin for Postpartum Haemorrhage Prevention in Low-Resource Settings: A Rapid Scoping Review
Source: Int J Environ Res Public Health. 2022 Mar 22;19(7):3765. doi: 10.3390/ijerph19073765 (PMC8998030; doi:10.3390/ijerph19073765)
Supplement: Supplementary file 1 [file ijerph-19-03765-s001.zip › ijerph-1641262-supplementary.pdf]

Supplementary Materials

1. Preferred Reporting Items for Systematic reviews and Meta-Analyses extension for Scoping Reviews (PRISMA-ScR) Checklist

SECTION	ITEM	PRISMA-ScR CHECKLIST ITEM	REPORTED ON PAGE #
**TITLE**			
Title	1	Identify the report as a scoping review.	1
**ABSTRACT**			
Structured summary	2	Provide a structured summary that includes (as applicable): background, objectives, eligibility criteria, sources of evidence, charting methods, results, and conclusions that relate to the review questions and objectives.	1
**INTRODUCTION**			
Rationale	3	Describe the rationale for the review in the context of what is already known. Explain why the review questions/objectives lend themselves to a scoping review approach.	2
Objectives	4	Provide an explicit statement of the questions and objectives being addressed with reference to their key elements (e.g., population or participants, concepts, and context) or other relevant key elements used to conceptualize the review questions and/or objectives.	2
**METHODS**			
Protocol and registration	5	Indicate whether a review protocol exists; state if and where it can be accessed (e.g., a Web address); and if available, provide registration information, including the registration number.	Yes, but not registered
Eligibility criteria	6	Specify characteristics of the sources of evidence used as eligibility criteria (e.g., years considered, language, and publication status), and provide a rationale.	Table 1
Information sources*	7	Describe all information sources in the search (e.g., databases with dates of coverage and contact with authors to identify additional sources), as well as the date the most recent search was executed.	3
Search	8	Present the full electronic search strategy for at least 1 database, including any limits used, such that it could be repeated.	See MEDLINE search below
Selection of sources of evidence†	9	State the process for selecting sources of evidence (i.e., screening and eligibility) included in the scoping review.	3
Data charting process‡	10	Describe the methods of charting data from the included sources of evidence (e.g., calibrated forms or forms that have been tested by the team before their use, and whether data charting was done	4

SECTION	ITEM	PRISMA-ScR CHECKLIST ITEM	REPORTED ON PAGE #
		independently or in duplicate) and any processes for obtaining and confirming data from investigators.	
Data items	11	List and define all variables for which data were sought and any assumptions and simplifications made.	3
Critical appraisal of individual sources of evidence§	12	If done, provide a rationale for conducting a critical appraisal of included sources of evidence; describe the methods used and how this information was used in any data synthesis (if appropriate).	4
Synthesis of results	13	Describe the methods of handling and summarizing the data that were charted.	4
**RESULTS**			
Selection of sources of evidence	14	Give numbers of sources of evidence screened, assessed for eligibility, and included in the review, with reasons for exclusions at each stage, ideally using a flow diagram.	Figure 1
Characteristics of sources of evidence	15	For each source of evidence, present characteristics for which data were charted and provide the citations.	Table 2<br>Table 3
Critical appraisal within sources of evidence	16	If done, present data on critical appraisal of included sources of evidence (see item 12).	n/a
Results of individual sources of evidence	17	For each included source of evidence, present the relevant data that were charted that relate to the review questions and objectives.	Table 2<br>Table 3
Synthesis of results	18	Summarize and/or present the charting results as they relate to the review questions and objectives.	Table 2
**DISCUSSION**			
Summary of evidence	19	Summarize the main results (including an overview of concepts, themes, and types of evidence available), link to the review questions and objectives, and consider the relevance to key groups.	16
Limitations	20	Discuss the limitations of the scoping review process.	18
Conclusions	21	Provide a general interpretation of the results with respect to the review questions and objectives, as well as potential implications and/or next steps.	18
**FUNDING**			
Funding	22	Describe sources of funding for the included sources of evidence, as well as sources of funding for the scoping review. Describe the role of the funders of the scoping review.	18

JBI = Joanna Briggs Institute; PRISMA-ScR = Preferred Reporting Items for Systematic reviews and Meta-Analyses extension for Scoping Reviews.

* Where *sources of evidence* (see second footnote) are compiled from, such as bibliographic databases, social media platforms, and Web sites.

† A more inclusive/heterogeneous term used to account for the different types of evidence or data sources (e.g., quantitative and/or qualitative research, expert opinion, and policy documents) that may be eligible in a scoping review as opposed to only studies. This is not to be confused with *information sources* (see first footnote).
‡ The frameworks by Arksey and O'Malley (6) and Levac and colleagues (7) and the JBI guidance (4, 5) refer to the process of data extraction in a scoping review as data charting.
§ The process of systematically examining research evidence to assess its validity, results, and relevance before using it to inform a decision. This term is used for items 12 and 19 instead of "risk of bias" (which is more applicable to systematic reviews of interventions) to include and acknowledge the various sources of evidence that may be used in a scoping review (e.g., quantitative and/or qualitative research, expert opinion, and policy document).

From: Tricco AC, Lillie E, Zarin W, O'Brien KK, Colquhoun H, Levac D, et al. PRISMA Extension for Scoping Reviews (PRISMAScR): Checklist and Explanation. Ann Intern Med. 2018;169:467–473. doi: 10.7326/M18-0850.

2. Protocol

Background and Objective

Excessive bleeding after delivery or postpartum hemorrhage (PPH) is a major cause of maternal death worldwide.[1] It overwhelmingly impacts the least developed nations, which account for 44% of maternal fatalities worldwide.[2]

Uterine atony causes around two-thirds of PPH cases. Therefore, the World Health Organization (WHO) recommends the administration of a prophylactic uterotonic to all women directly after birth to prevent PPH. This is the most critical component of the active management of the third stage of labor, and oxytocin is the uterotonic of choice according to WHO recommendations.[3-5]

Heat-stable carbetocin (HSC), a long-acting synthetic oxytocin analog recommended only for PPH prevention, was recently added to the core list of reproductive health medicines of the WHO Model List of Essential Medicines (2019 edition).[6] HSC does not require cold-chain transport and storage that impede oxytocin use—an operational advantage in low-resource settings. Several studies and systematic reviews have demonstrated the effectiveness of HSC, including in low-income countries.

HSC has been relatively costly compared to other uterotonics. Recent subsidization efforts for the public sector of low-income and middle-income countries have resulted in competitive prices. The 2021 Product Catalogue of the United Nations Population Fund (UNFPA) listed a price of USD 0.413 per HSC ampoule compared to USD 0.334 per oxytocin ampoule.[7] Against this background, the WHO recommends HSC in situations when (i) oxytocin is unavailable or of dubious quality, (ii) there is no cold transportation and storage capability, (iii) its cost is comparable to that of other effective uterotonics, and (iv) there is skilled health personnel to inject it.[3]

To facilitate the implementation of HSC in low-resource settings, policymakers and program managers need additional information beyond its effectiveness. This includes the feasibility of its use, its acceptability by women and providers, and other health system requirements, such as policy change, staff capacitation, or procurement and storage considerations. A synthesis of existing experience from low-resource settings may offer helpful guidance. As such, this study aims to summarize HSC implementation experience from low-resource settings.

Methods

We will conduct a rapid scoping review of the literature to synthesize recent practices and health system considerations to inform HSC roll-out in low-resource

settings. A rapid review is a type of knowledge synthesis in which researchers abridge or skip parts of the systematic review process to obtain information in a shortened timeframe.[8] Typically, the quality of included reports is not appraised as scoping reviews are intended to give researchers, decision-makers, and practitioners an overview of a topic to identify the main ideas, evidence types, and knowledge gaps within an emerging topic.[9] This rapid scoping review is commissioned by UNFPA with an 8-week timeline and will be carried out by one reviewer. The PRISMA statement will be used to report the results.[10]

Search Strategy

We will use the methodologically rigorous rapid scoping review approach described in the Cochrane Handbook for Systematic Reviews. We will search the following five bibliographic databases via Ovid to find relevant documents: MEDLINE, EMBASE, Emcare, the Joanna Briggs Institute Evidence-Based Practice (JBI EBP) Database, and the Maternity and Infant Care Database (MIDIRS). We will also search the Cochrane Library. The leading search concepts will be carbetocin, postpartum hemorrhage, and developing countries. The search will be limited to documents in English, French, and Spanish published between 1 January 2011 and 15 September 2021.

Study Selection: Screening

Titles and abstracts will be reviewed at the first stage of screening. Potential papers will be retrieved and evaluated for inclusion using the criteria in Table 1, which will guide the final selection of full-text publications. The eligibility criteria are defined using the population, concept, and context (PCC) framework in addition to study design. The PCC framework is recommended by the JBI for Scoping Reviews as a more suitable alternative to the PICO mnemonic (population, intervention, comparator, and outcome) recommended for systematic reviews.[11 12]

Table 1. Eligibility criteria

Study design	Randomized controlled trials; non-randomized trials
Timeline	Published between 1 January 2011 and 15 September 2021
P (population)	Women who had a vaginal or cesarean birth
C (concept)	Postpartum hemorrhage; feasibility; acceptability; health system considerations
C (context)	Low-income countries; lower-middle-income countries

Data Abstraction

Non-duplicated articles will be imported into JBI SUMARI (System for the Unified Management, Assessment, and Review of Information) for further screening.[13] Titles and abstracts will be assessed for eligibility, and the full texts of eligible articles

reviewed. The data of included studies will be extracted using tables developed a priori and pilot-tested on a random sample of articles. We will use acceptability, feasibility, and health system considerations drawn from the WHO framework of the six-health system building blocks as lenses to extract data. The building blocks comprise (i) government and policy alignment, (ii) procurement channels and commodity security, (iii) health staff awareness, motivation, and training, (iv) service delivery, (v) health information system, and (vi) financing.[14]

Synthesis

The review findings will be presented narratively with the aid of tables on study characteristics and relevant program considerations. We will describe the reports included.

3. World Bank low-income and lower-middle-income countries

Sources: https://datahelpdesk.worldbank.org/knowledgebase/articles/906519-world-bank-country-and-lending-groups.

LOW-INCOME ECONOMIES ($1,045 OR LESS)

Afghanistan	Guinea-Bissau	Somalia
Burkina Faso	Korea, Dem. People's Rep	South Sudan
Burundi	Liberia	Sudan
Central African Republic	Madagascar	Syrian Arab Republic
Chad	Malawi	Togo
Congo, Dem. Rep	Mali	Uganda
Eritrea	Mozambique	Yemen, Rep.
Ethiopia	Niger	
Gambia, The	Rwanda	
Guinea	Sierra Leone	

LOWER-MIDDLE INCOME ECONOMIES ($1,046 TO $4,095)

Angola	Honduras	Philippines
Algeria	India	Samoa
Bangladesh	Indonesia	São Tomé and Principe
Belize	Iran, Islamic Rep	Senegal
Benin	Kenya	Solomon Islands
Bhutan	Kiribati	Sri Lanka
Bolivia	Kyrgyz Republic	Tanzania
Cabo Verde	Lao PDR	Tajikistan
Cambodia	Lesotho	Timor-Leste
Cameroon	Mauritania	Tunisia
Comoros	Micronesia, Fed. Sts.	Ukraine
Congo, Rep.	Mongolia	Uzbekistan
Côte d'Ivoire	Morocco	Vanuatu
Djibouti	Myanmar	Vietnam
Egypt, Arab Rep.	Nepal	West Bank and Gaza
El Salvador	Nicaragua	Zambia
Eswatini	Nigeria	Zimbabwe
Ghana	Pakistan	
Haiti	Papua New Guinea	

4. MEDLINE search strategy

1 carbetocin.mp.
2 Postpartum Hemorrhage/
3 (delayed postpartum hemorrhage or hemorrhage, delayed postpartum or hemorrhage, immediate postpartum or hemorrhage, postpartum or immediate postpartum hemorrhage or postpartum hemorrhage or postpartum hemorrhage, delayed or postpartum hemorrhage, immediate).mp.
4 2 or 3
5 Developing Countries/
6 (countries, developing or countries, least developed or countries, less-developed or countries, third-world or countries, under-developed or country, developing or country, least developed or country, less-developed or country, third-world or country, under-developed or developed countries, least or developed country, least or developing countries or developing country or developing nation or developing nations or least developed countries or least developed country or less developed countries or less developed nations or less-developed countries or less-developed country or less-developed nation or less-developed nations or nation, less-developed or nation, third-world or nation, under-developed or nations, developing or nations, less-developed or nations, third-world or nations, under-developed or third world countries or third world nations or third-world countries or third-world country or third-world nation or third-world nations or under developed countries or under developed nations or under-developed countries or under-developed country or under-developed nation or under-developed nations).mp.
7 (Angola or Algeria or Bangladesh or Belize or Benin or Bhutan or Bolivia or cabo verde or cape verde or "republic of cape verde" or Cambodia or Cameroon or Comoros or "congo (brazzaville)" or "republic of the congo" or cote d'ivoire or ivory coast or "republic of cote diivoire" or Djibouti or "arab republic of egypt" or egypt or El Salvador or Eswatini or Ghana or Haiti or Honduras or India or Indonesia or iran or "islamic republic of iran" or Kenya or Kiribati or kirghiz ssr or kirghizia or kirgizstan or kyrgyz republic or kyrgyzstan or lao pdr or laos or Lesotho or micronesia or "micronesia, federated states of" or Mauritania or Mongolia or Morocco or Myanmar or Nepal or Nicaragua or Nigeria or Pakistan or Papua New Guinea or Philippines or Samoa or "sao tome and principe" or Senegal or Solomon Islands or Sri Lanka or Tanzania or Tajikistan or "democratic republic of timor-leste" or east timor or timor-leste or Tunisia or Ukraine or Uzbekistan or Vanuatu or Vietnam or "Gaza Strip (Palestine)" or "West Bank and Gaza" or gaza strip or west bank or Zambia or Zimbabwe):ti,ab,kw (Word variations have been searched)
8 5 or 6 or 7

9 1 and 4 and 8
